# Serum miRNA125a-5p, miR-125b-5p, and miR-433-5p as biomarkers to differentiate between posterior circulation stroke and peripheral vertigo

**DOI:** 10.1186/s12883-020-01946-3

**Published:** 2020-10-10

**Authors:** Naruchorn Kijpaisalratana, Pattaraporn Nimsamer, Ariya Khamwut, Sunchai Payungporn, Trairak Pisitkun, Aurauma Chutinet, Nattawan Utoomprurkporn, Stephen J. Kerr, Pakkawan Vongvasinkul, Nijasri C. Suwanwela

**Affiliations:** 1 Division of Neurology, Department of Medicine, Faculty of Medicine, Chulalongkorn University, Rama IV Road, Bangkok, 10330 Thailand; 2 Division of Academic Affairs, Faculty of Medicine, Chulalongkorn University, Bangkok, Thailand; 3 Chula Neuroscience Center, King Chulalongkorn Memorial Hospital, Thai Red Cross Society, Bangkok, Thailand; 4 Center of Excellence in Systems Biology, Research Affairs, Faculty of Medicine, Chulalongkorn University, Bangkok, Thailand; 5 Department of Biochemistry, Faculty of Medicine, Chulalongkorn University, Bangkok, Thailand; 6 Chulalongkorn Stroke Center, King Chulalongkorn Memorial Hospital, Thai Red Cross Society, Bangkok, Thailand; 7 Otoneurology Unit, Otolaryngology Department, Faculty of Medicine, Chulalongkorn University, King Chulalongkorn Memorial Hospital, Bangkok, Thailand; 8 UCL Ear Institute, Faculty of Brain Science, University College London, London, UK; 9 Research Affairs, Faculty of Medicine, Chulalongkorn University, Bangkok, Thailand; 10 The HIV Netherlands Australia Thailand Research Collaboration (HIV-NAT), The Thai Red Cross AIDS Research Centre, Bangkok, Thailand; 11 The Kirby Institute, The University of New South Wales, Sydney, Australia

**Keywords:** Acute vertigo, Stroke biomarker, microRNA, Posterior circulation stroke, Central vertigo, Peripheral vertigo

## Abstract

**Background:**

Acute vertigo is a common presentation of inner ear disease. However, it can also be caused by more serious conditions, especially posterior circulation stroke. Differentiating between these two conditions by clinical presentations and imaging studies during the acute phase can be challenging. This study aimed to identify serum microRNA (miRNA) candidates that could differentiate between posterior circulation stroke and peripheral vertigo, among patients presenting with acute vertigo.

**Methods:**

Serum levels of six miRNAs including miR-125a-5p, miR-125b-5p, miR-143-3p, miR-342-3p, miR-376a-3p, and miR-433-5p were evaluated. Using quantitative reverse-transcription polymerase chain reaction (RT-qPCR), the serum miRNAs were assessed in the acute phase and at a 90 day follow-up visit.

**Results:**

A total of 58 patients with posterior circulation stroke (*n* = 23) and peripheral vertigo (*n* = 35) were included in the study. Serum miR-125a-5p (*P* = 0.001), miR-125b-5p (*P* <  0.001), miR-143-3p (*P* = 0.014) and miR-433-5p (*P* = 0.0056) were present at significantly higher levels in the acute phase, in the patients with posterior circulation infarction. Based on the area under the receiver operating characteristic curve (AUROC) only miR-125a-5p (0.75), miR-125b-5p(0.77), and miR-433-5p (0.71) had an acceptable discriminative ability to differentiate between the central and peripheral vertigo. A combination of miRNAs revealed no significant improvement of AUROC when compared to single miRNAs.

**Conclusion:**

This study demonstrated the potential of serum miR-125a-5p, miR-125b-5p, and miR-433-5p as biomarkers to assist in the diagnosis of posterior circulation infarction among patients presenting with acute vertigo.

## Background

Acute vertigo is a common presentation of inner ear disease. However, it can also be caused by more serious conditions, particularly posterior circulation stroke. Differentiating between these two conditions during the acute phase of vertigo on the basis of clinical presentations and imaging studies can be challenging. Although a combination of bedside physical examinations consisting of head impulse test, nystagmus, and test of skew (HINTS) has both high sensitivity and specificity for differentiating posterior circulation stroke from peripheral vertigo [1], the test is not applicable in most patients especially after vestibular symptoms have resolved [2]. Computed tomography (CT) is not sensitive for diagnosing posterior circulation stroke [3]. Although magnetic resonance imaging (MRI) has higher sensitivity, initial MRI done within the first 48 h can miss the infarctions in 12% of patients [1].

MicroRNA (miRNA) is a small non-coding ribonucleic acid containing 20–24 nucleotides. MiRNA regulates protein synthesis which is a biological stage and organ-specific [4]. It is stable in various body fluids, a factor contributing to its potential as a biomarker [5, 6]. MiRNAs have been demonstrated to have prognostic, diagnostic, and therapeutic biomarker properties among stroke patients [7].

### Aims

Due to the diagnostic challenge in patients presenting with acute vertigo, this study aimed to determine the potential of miRNA as a biomarker to differentiate between central vertigo due to posterior circulation infarction and peripheral vertigo. Serum levels of 6 miRNAs including 3 previously reported miRNAs: miR-125a-5p, miR-125b-5p, miR-143-3p [8] and 3 miRNAs identified in a pilot study by NanoString nCounter Analysis (Supplementary material): miR-342-3p, miR-376a-3p, and 433-5p were evaluated in patients presenting with acute vertigo.

## Methods

### Study design and study population

This is a single-center prospective cohort study conducted between June 2017–December 2019. The study was approved by the Institutional Review Board of the Faculty of Medicine, Chulalongkorn University (IRB No. 131/60). Written informed consent was obtained from every patient prior to the enrollment. Patients aged ≥45 years old with acute vertigo who presented to the emergency department of King Chulalongkorn Memorial Hospital within 72 h after symptom onset were consecutively screened for eligibility. Exclusion criteria were patients with altered consciousness, body temperature > 100.4 °F, white blood cell count > 15,000 cell/μL, serum creatinine > 2 mg/dl, liver enzymes > 3 fold of upper normal limit or cirrhosis, known history of active malignancy or autoimmune disease, and concurrent intracranial pathology other than acute ischemic stroke.

### Diagnosis of posterior circulation stroke

The final diagnosis of central vertigo due to ischemic stroke was made by a neurologist and confirmed by the presence of restricted diffusion in the diffusion-weighted images on MRI. MRI studies were performed using a 3-Tesla scanner (Skyra, Siemens, Erlangen, Germany). Ischemic stroke severity was assessed according to the National Institute of Health Stroke Scale (NIHSS) by a trained neurologist. The cerebral infarction volume was quantified by the diffusion weighted images on 3-Tesla MRI, using Olea Sphere™ Version 3.0 software (Olea Medical®, La Ciotat, France).

### Diagnosis of peripheral vertigo

Peripheral vertigo was diagnosed by an otolaryngologist according to the diagnostic criteria of benign paroxysmal positional vertigo (BPPV) [9] and Meniere’s disease [10]. The diagnosis of vestibular neuritis was made by agreement between both neurologist and otolaryngologist, and confirmed by absence of any evidence of cerebral infarction on MRI. MRI was performed in all patients whose clinical not compatible with the diagnostic criteria of BPPV and Meniere’s disease. In case of negative MRI study, they were classified as “MRI-negative other peripheral vertigo”.

### Specimen collection and miRNA isolation

Blood was collected within 72 h after onset of acute vertigo upon arrival and at a follow-up visit on day 90. Venous blood was collected in tubes containing clot activator. After 30 min of coagulation, serum supernatant was separated by centrifugation at 1000 g, 4 °C, for 15 min, followed by an additional serum centrifugation at 2500 g, 4 °C, for 15 min to remove residual debris. Serum was frozen and stored at − 80 °C until used. MiRNAs were isolated from 200 μl of the serum using Geneaid® miRNA isolation kit (Geneaid Biotech Ltd., New Taipei City, Taiwan) according to the manufacturer’s protocol.

### RT-qPCR

The miRNA candidates were quantified by RT-qPCR. First, the miRNA was polyuridylated by poly(U) polymerase (New England BioLabs®, Ipswich, MA, USA). Then, the cDNA molecules were reverse transcribed by the universal poly(A) stem-loop RT primers. Finally, the miRNA quantification was performed by real-time PCR using the SYBR® Green fluorescence (New England BioLabs®, Ipswich, MA, USA) [11]. Absolute quantification of the target miRNAs in serum sample was performed by the standard curve method. A standard curve was generated by serial dilution of a known concentration of the target miRNAs. Quantification of miRNA level was performed by laboratory staff blinded to clinical presentation and neuroimaging findings.

### Statistical analysis

Visual inspection of graphs and the Kolmogorov-Smirnov test was used to determine whether data was normally distributed. Continuous variables were presented as mean ± standard deviation (SD) or median with interquartile range (IQR), as appropriate. Unpaired-t tests or Mann-Whitney U tests were used to formally compare study group characteristics between central and peripheral vertigo groups, for data with continuous normal and non-normal distributions, respectively. Categorical variables were presented as frequencies and percentages. Mann-Whitney U tests were used to compare miRNA levels between groups at each timepoint. Paired within group comparisons of non-normal data were made using a Wilcoxon signed rank test. Spearman’s rho was used to assess correlations between miRNA levels and ischemic stroke severity by NIHSS and infarct volume. Receiver operating characteristic (ROC) curves were generated to demonstrate the discriminative ability of the biomarkers to distinguish between central and peripheral vertigo groups. Binary logistic regression was performed to assess the association between miRNAs and stroke, after adjusting for potential confounders, that were imbalanced between study group. We used Youden’s index to determine cutoffs defining the maximum potential effectiveness of the miRNAs. Missing data at the follow-up period on day 90 were excluded from analysis. Statistical analysis was performed by SPSS 22.0 for Mac software package (SPSS, Inc., sChicago, IL, USA) and STATA 15 for windows (StataCorp, LLC., College Station, TX, USA). Graphs demonstrating biomarker levels were generated by Prism 8 for OS X (GraphPad Software, Inc., San Diego, CA, USA).

## Results

### Patient characteristics

A total of 58 patients, 23 with central vertigo due to posterior circulation stroke and 35 with peripheral vertigo were studied. Patients with posterior circulation stroke included cerebellar infarction (52%), brainstem infarction (26%), and both cerebellar and brainstem infarction (22%). In peripheral vertigo group, there were patients with BPPV (49%), Meniere’s Disease (11%), vestibular neuritis (11%), and MRI-negative other peripheral vertigo (29%). Most of the baseline characteristics among 2 groups were comparable with the exception of longer onset to blood collection time, higher number of females, diabetics, patients with previous myocardial infarction, and current smokers among those with posterior circulation stroke. (Table 1). No recruited patients received low/high molecular weight heparin prior to blood sampling for miRNA analysis.

**Table 1:** Baseline characteristics, laboratory parameters, and medication in acute vertigo patients

	Central Vertigo (*n* = 23)	Peripheral Vertigo (*n* = 35)	*P* value
Age, years, mean (SD)	64.52 (11.80)	63.69 (9.42)	0.766
Female, % (n)	21.7% (5)	82.9% (29)	0.001*
Body mass index, kg/m^2^, mean (SD)	24.89 (3.96)	24.56 (4.62)	0.59
Onset to blood collection (hrs), mean (SD)	37.59 (16.94)	21.35 (17.91)	0.001*
Stroke risk factors, % (n)			
- Hypertension	65.2% (15)	51.4% (18)	0.30
- Diabetes mellitus	52.2% (12)	17.1% (6)	0.005*
- Dyslipidemia	56.5% (13)	48.6% (17)	0.55
- History of stroke	13.0% (3)	28.6% (10)	0.17
- History of myocardial infarction	21.7% (5)	2.9% (1)	0.02*
- Smoking	26.1% (6)	0	0.001*
Previous history of vertigo, % (n)	13% (3)	31.4% (11)	0.11
Systolic BP, mmHg, med (IQR)	161 (146–170)	149 (139–168)	0.10
Diastolic BP, mmHg, med (IQR)	90 (75–100)	80 (68–90)	0.14
Pulse rate, bpm, med (IQR)	80 (60–85)	76 (70–82)	0.87
Laboratory parameters			
- Hemoglobin, g/dL, med (IQR)	13.7 (13.1–15.4)	13.0 (12.5–13.4)	0.009*
- WBC count, *10^3^/μL, mean (SD)	9.91 (2.95)	8.29 (2.21)	0.03*
- Platelets, *10^3^/μ, mean (SD)	227.61 (66.43)	266.24 (81.14)	0.07
- Creatinine, mg/dL, med (IQR)	0.84 (0.71–0.98)	0.67 (0.61–0.82)	0.04*
- Fasting blood sugar, mg/dL, med (IQR)	130.0 (103–154)	100.0 (80–113.5)	0.008*
- HbA_1_C, %, med (IQR)	6.3 (5.6–7.2)	5.6 (5.28–6.2)	0.02*
Medication, n(%)			
- Antiplatelets	17.4% (4)	37.1% (13)	0.11
- Anticoagulant	0% (0)	2.9% (1)	0.31

*WBC* white blood cell, *LDL* low density lipoprotein, *HDL* high density lipoprotein, *SGOT* serum glutamic-oxaloacetic transaminase, *SGPT* serum glutamic pyruvic transaminase, *ALP* alkaline phosphatase, *ACEI* angiotensin-converting enzyme inhibitor, *ARBs* angiotensin II receptor blockers

*P <  0.05

### Clinical presentations

The signs and symptoms of spinning, sense of imbalance, nausea and vomiting, oscillopsia, and nystagmus in both groups were indistinguishable. Physical signs including skew deviation, diplopia, and limb ataxia were found exclusively in the patients with central vertigo. A positive HINTS test was found only amongst 4 (11.4%) of peripheral vertigo patients. However, this was not statistically significant (Table 2).

**Table 2:** Clinical presentations of acute vertigo

Clinical presentations [%(n)]	Central Vertigo (*n* = 23)	Peripheral Vertigo (*n* = 35)	*P* value
Sense of spinning	65.2% (15)	82.9% (29)	0.13
Sense of imbalance	95.7% (22)	88.6% (31)	0.35
Nausea/vomiting	60.9% (14)	77.1% (27)	0.18
Positive HINTS	0% (0)	11.4% (4)	0.09
Nystagmus	47.8% (11)	54.4% (19)	0.63
Bilateral direction changing	17.4% (4)	0% (0)	
Vertical	4.3% (1)	0% (0)	
Torsional	0% (0)	0% (0)	
Unidirectional horizontal	13.0% (3)	17.1% (6)	
Multidirectional	13.0% (3)	0% (0)	
Diagnostic maneuver	0% (0)	37.0% (13)	
Skew deviation	13.0% (3)	0% (0)	0.03*
Diplopia	30.4% (7)	0% (0)	0.001*
Oscillopsia	8.7% (2)	8.6% (3)	0.99
Limb ataxia	18 (78.3%)	0% (0)	< 0.001*

*HINTS* head impulse test, nystagmus, test of skew deviation

*P < 0.05

### Serum miRNAs expression level

**Fig. 1:**
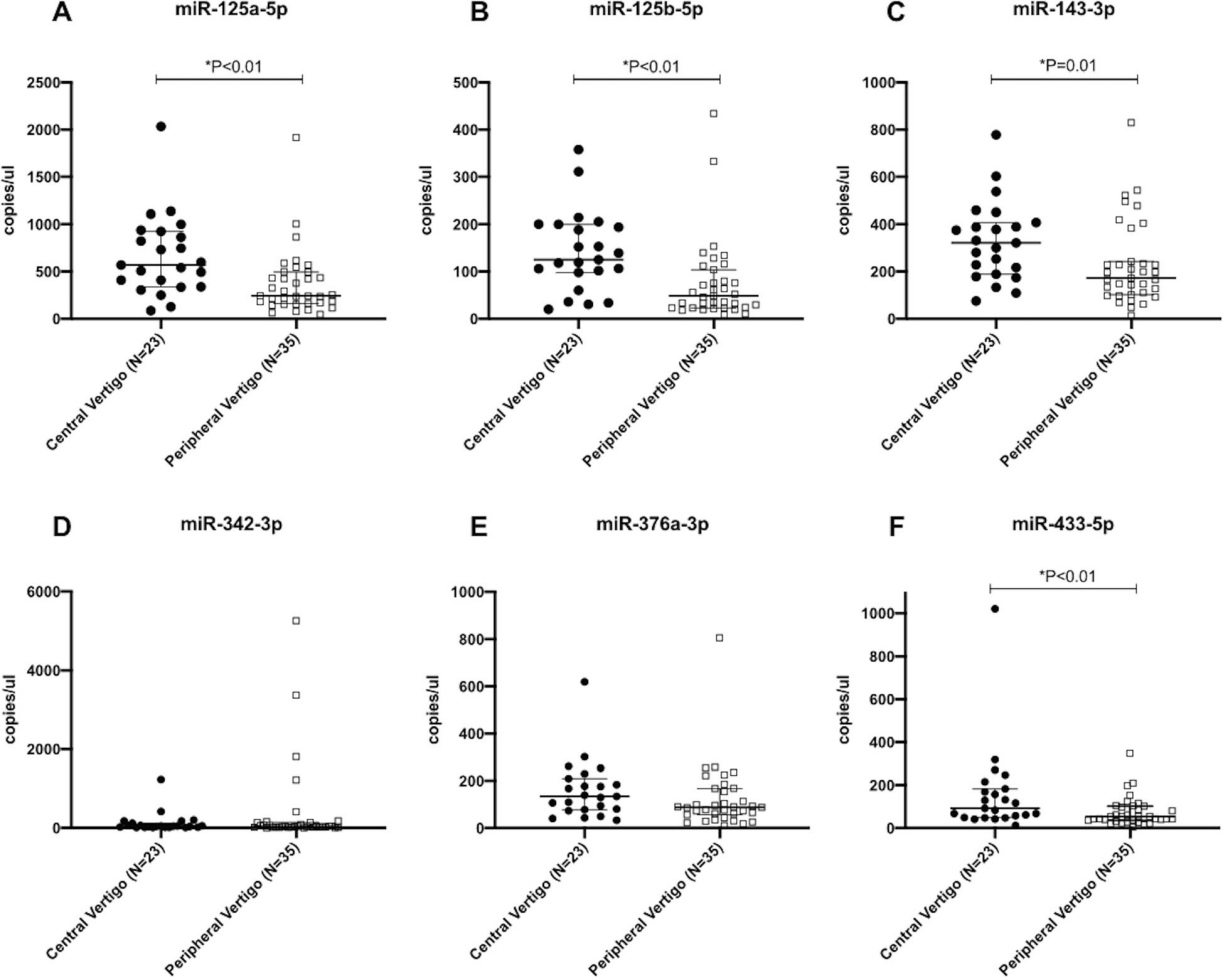
Serum miRNA levels in patients presenting with acute vertigo

During the acute phase, 4 out of 6 selected miRNAs including miR-125a-5p, miR-125b-5p, miR-143-3p, and miR-433-5p were present at significantly higher level among patients with central vertigo due to posterior circulation infarction. (Table 3, Fig. 1).

**Table 3:** Biomarker level during acute phase in patients with central and peripheral vertigo

miRNA level [median (IQR)]	Central Vertigo (*n* = 23)	Peripheral Vertigo (*n* = 35)	*P* value
miR-125a-5p	568.7 (338.2–924.5)	244.5 (160.8–495.1)	0.001*
miR-125b-5p	125.0 (98.01–199.8)	48.6 (22.66–103.7)	< 0.001*
miR-143-3p	321.6 (188.7–407.2)	172.5 (102.2–242.2)	0.014*
miR-342-3p	44.8 (16.39–118.7)	37.76 (14.57–136.4)	0.81
miR-376a-3p	134.3 (78.07–209.5)	87.93 (56.63–167.6)	0.056
miR-433-5p	92.13 (49.06–183.2)	53.45 (35.37–102.3)	0.006*

*P < 0.05

We assessed the correlation between distinct miRNA levels using Spearman’s rank correlation. All showed significant correlations (*P* < 0.001) with Spearman’s rho ranging from 0.4403–0.8472, with the exception of miR342-3p which only showed a significant correlation with miR-376a-3p. (Supplementary Table 1).

**Fig. 2:**
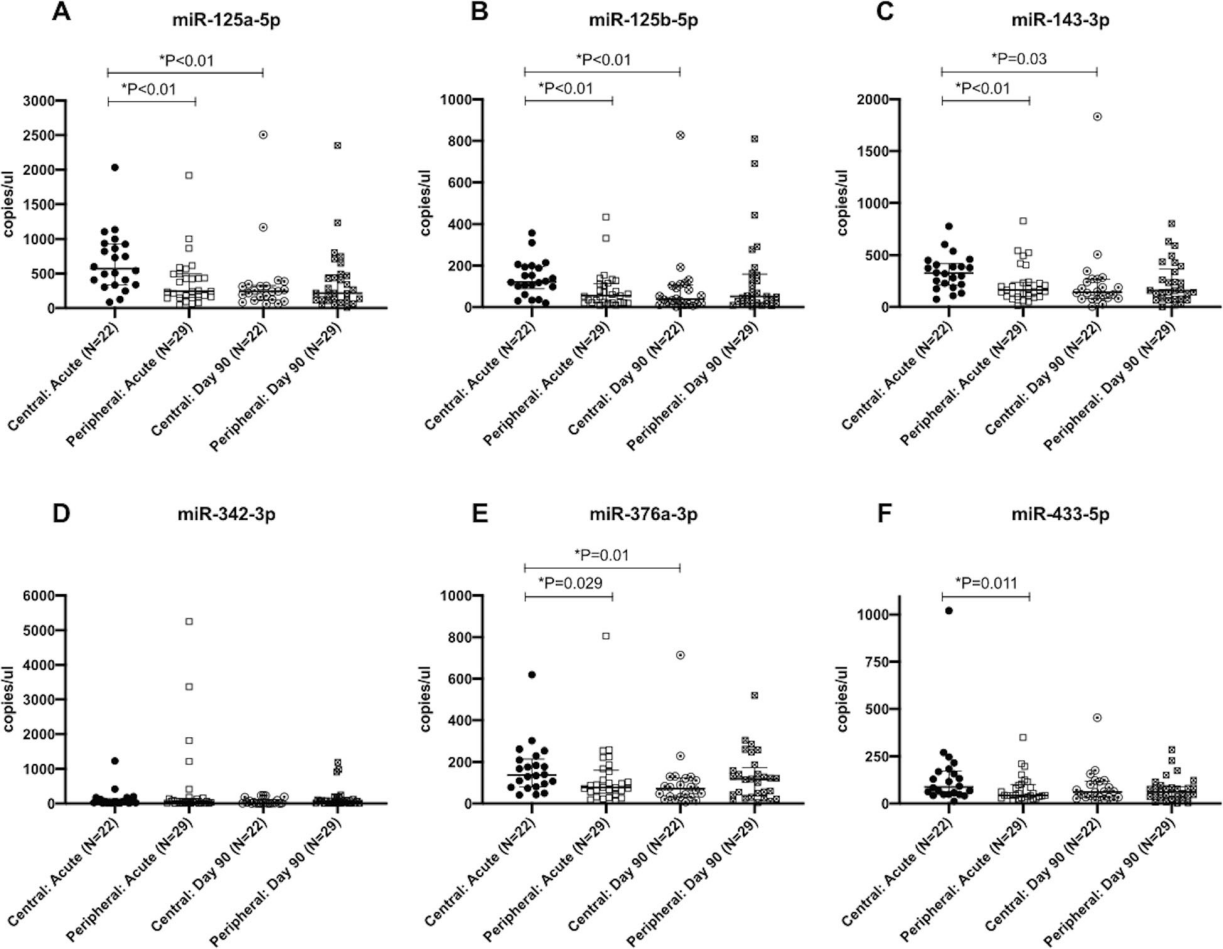
Temporal expression profile of miRNA levels in central and peripheral vertigo

In the temporal expression profile study, serum miRNA levels were measured in the acute phase and on day 90. One patient in the central vertigo group and 6 patients in the peripheral vertigo group were lost to follow-up on day 90. The levels of miR-125a-5p (median (IQR): central: 569.9 (337.2–927.8) copies/μL vs. peripheral 244.5 (156.0–496.6) copies/μL, *P* = 0.004), miR-125b-5p (central: 122.2 (88.56–195.3) copies/μL vs. peripheral: 56.0 (22.6–113.9) copies/μL, *P* = 0.003), miR-143-3p (central: 326.4 (210.1–418.1) copies/μL vs. peripheral: 165.4 (100.3–238.0) copies/μL, *P* = 0.007), miR-376a-3p (central: 136.8 (80.1–214.5) copies/μL vs. peripheral: 77.9 (50.0–160.8) copies/μL, *P* = 0.029), and miR-433-5p (central: 88.1 (48.9–172.3) copies/μL vs. peripheral: 44.0 (33.0–101.1) copies/μL, *P* = 0.011) were significantly higher during the acute phase in patients with central vertigo. The level of miR-125a-5p (acute: 569.9 (337.2–927.8) copies/μL vs. day 90: 242.4 (120.0–326.9) copies/μL, *P* = 0.008); miR-125b-5p (acute: 122.2 (88.56–195.3) copies/μL vs. day 90: 38.2 (14.02–106.4) copies/μL, *P* = 0.003); miR-143-3p (acute: 326.4 (210.1–418.1) copies/μL vs. day 90: 141.9 (81.03–268.3) copies/μL, *P* = 0.03), and miR-376a-3p (acute: 136.8 (80.1–214.5) copies/μL vs. day 90: 71.7 (30.8–123.3) copies/μL, *P* = 0.01) were significantly decreased by day 90 in stroke patients. In the peripheral vertigo patients, there were no significant differences in the levels of all 6 miRNAs between the acute phase and day 90 (Fig. 2).

Binary logistic regression was performed to determine associations between serum miRNAs and diagnosis of posterior circulation stroke. In both univariate and multivariate analyses after adjusting for sex, onset to blood collection time, diabetes mellitus, history of myocardial infarction, and smoking, serum miR-125a-5p, miR-125b-5p, miR-143-3p, and miR-433-5p were associated with the diagnosis of stroke (Supplementary Table 2).

### Relationship of miRNAs level with ischemic stroke severity

The median (IQR) stroke severity determined by NIHSS in the patients with acute vertigo due to posterior circulation infarction was 3 (2-6). There was no correlation between the NIHSS and the serum level of miRNAs (Supplementary Table 3). The median (IQR) volume of infarction was 4.51 (0.66–16.26) ml. Serum miR-125a-5p and miR-376a-3p demonstrated weak positive correlation with the cerebral infarction volume which remained significant after correction for multiple testing (Supplementary Table 4). In addition to stroke severity, there was no correlation between the miRNA level and the onset to blood collection time among patients with posterior circulation infarction (Supplementary Table 5).

### Diagnostic ability of serum miRNA in acute vertigo

The areas under the ROC curve (95%CI) of the six serum miRNAs are demonstrated in Table 4. Based on the AUROC and 95% CI, only miR-125a-5p, miR-125b-5p, and miR-433-5p had an acceptable ability to differentiate between the central and peripheral vertigo. All possible combination of these 3 miRNAs including miR-125a-5p, miR-125b-5p, and miR-433-5p were examined and revealed no statistically improvement of the discriminative ability (Supplementary Table 6 and Supplementary Figure 2). Optimal cut off levels determined by Youden’s index were: miR-125a-5p: 299 copies/μL (sensitivity: 87.0%; specificity: 57.1%); miR-125b-5p: 89 copies/μL (sensitivity: 78.3%; specificity: 74.3%); miR-433-5p: 46 copies/μL (sensitivity: 87%; specificity: 48.6%).

**Table 4:** AUROC of the miRNAs for diagnosis of central vertigo

	AUROC	95% CI
miR-125a-5p	0.7516	0.6196–0.8835
miR-125b-5p	0.7689	0.6418–0.8961
miR-143-3p	0.6919	0.5523–0.8315
miR-342-3p	0.5193	0.3674–0.6712
miR-376a-3p	0.6497	0.5042–0.7952
miR-433-5p	0.7143	0.5780–0.8506
miR-125a-5p + miR-125b-5p	0.7665	0.6382–0.8948
miR-125a-5p + miR-433-5p	0.7503	0.6171–0.8835
miR-125b-5p + miR-433-5p	0.7590	0.6282–0.8899
miR-125a-5p + miR-125b-5p + miR-433-5p	0.7627	0.6345–0.8910

## Discussion

Serum levels of miR-125a-5p, miR-125b-5p, miR-143-3p, and miR-433-5p were present at significantly higher levels among patients with central vertigo due to posterior circulation infarction in both the acute phase and temporal expression profile studies. However, based on AUROC analysis, only miR-125a-5p, miR-125b-5p, and miR-433-5p demonstrated potential as posterior circulation stroke biomarkers for diagnosis of acute vertigo. Serum levels of miR-125a-5p and miR-125b-5p significantly decreased by day 90 among patients with posterior circulation infarction. These findings suggest that the elevation of both miR-125a-5p and miR-125b-5p occurred in the acute phase of ischemic stroke, and emphasize the capability of these miRNAs as posterior circulation stroke biomarkers. Interestingly, there were no significant differences in the expression level of serum miR-125a-5p, miR-125b-5p, and miR-433-5p between the patients with central and peripheral vertigo on day 90 and in the patients with peripheral vertigo between day 0 and day 90. This temporal expression pattern might be useful in elucidating the mechanism of miRNA involvement in the pathophysiology and pathogenesis of acute ischemic stroke. An elevation of serum miRNA in the acute phase of ischemic stroke is possibly due to the passive release from ischemia-vulnerable neurons [12] or by active secretion via extracellular vesicles [8, 13].

The sensitivity of serum miR-125a-5p, miR-125b-5p, and miR-433-5p to identify patients with posterior circulation stroke in our study was higher than that previously reported using CT scan as a diagnostic neuroimaging tool. Our study demonstrated low or no correlation between the level of serum miRNAs in ischemic stroke, and severity determined by both the NIHSS and infarction volume. Our findings are consistent with those previously reported by Tiedt et al. [8] which emphasized the potential of these biomarker candidates as adjuncts in the diagnosis of acute ischemic stroke regardless of stroke severity.

MiR-125a-5p and miR-125b-5p have previously shown associations with acute ischemic stroke from an RNA sequencing study [8]. An in vivo experiment demonstrated that miR-125a-5p regulates blood brain barrier function [14]. MiR-125b-5p has previously been shown to be a predictive biomarker candidate for ischemic stroke outcomes [15, 16]. MiR-433 is highly expressed in the brain tissue [11]. Upregulation of miR-433 inhibits cell migration and proliferation of human umbilical vein vascular endothelial cells and neurons by targeting the Hypoxia-Inducible Factor-1α (HIF-1α) [14]. Further study is warranted to evaluate the functions of these miRNAs, and their role in acute ischemic stroke pathophysiology.

Our study has limitations due to being conducted in a single-center, using a relatively small number of patients. An independent replication cohort is essential to validate the novel findings in this study.

## Conclusion

This study demonstrated the potential of serum miR-125a-5p, miR-125b-5p, and miR-433-5p as biomarkers for diagnosing posterior circulation infarction among patients presenting with acute vertigo.

## Supplementary information

**Additional file 1:** **Supplementary Method. Supplementary Result. Table 1.** Spearman’s correlation coefficients for each miRNA combination. **Table 2.** Univariate and multivariate logistic regression for diagnosis of stroke. **Table 3.** Spearman’s correlation coefficients for the association between miRNA levels and NIHSS. **Table 4.** Spearman’s correlation coefficients for the association between miRNA levels and infarction volume. **Table 5.** Spearman’s correlation coefficients for the association between miRNA level and onset to blood collection time. **Table 6.** *P* values of the AUROC comparison between individual miRNA levels and a combination of miRNAs. **Figure 1.** Venn diagram of potential miRNA candidates. **Figure 2.** AUROC of potential miRNA candidates.

## Data Availability

The datasets are available from the corresponding author upon reasonable request, after clearance by the institutional ethics committee.

## Abbreviations

miRNA: micro-Ribonucleic Acid
RT-qPCR: Quantitative reverse transcription polymerase chain reaction
AUROC: Area under the receiver operating characteristic curve
HINTS: Head impulse test, nystagmus, and test of skew
CT: Computed tomography
MRI: Magnetic resonance imaging
BPPV: Benign paroxysmal positional vertigo
NIHSS: National Institute of Health Stroke Scale
SD: Standard deviation
IQR: Interquartile range
