# Supplementary material for: Serum miRNA125a-5p, miR-125b-5p, and miR-433-5p as biomarkers to differentiate between posterior circulation stroke and peripheral vertigo
Source: BMC Neurol. 2020 Oct 10;20:372. doi: 10.1186/s12883-020-01946-3 (PMC7547489; doi:10.1186/s12883-020-01946-3)
Supplement: Supplementary file 1 — Additional file 1: Supplementary Method. Supplementary Result. Table 1. Spearman’s correlation coefficients for each miRNA combination. Table 2. Univariate and multivariate logistic regression for diagnosis of stroke. Table 3. Spearman’s correlation coefficients for the association between miRNA levels and NIHSS. Table 4. Spearman’s correlation coefficients for the association between miRNA levels and infarction volume. Table 5. Spearman’s correlation coefficients for the association between miRNA level and onset to blood collection time. Table 6. P values of the AUROC comparison between individual miRNA levels and a combination of miRNAs. Figure 1. Venn diagram of potential miRNA candidates. Figure 2. AUROC of potential miRNA candidates. [file 12883_2020_1946_MOESM1_ESM.docx]

**SUPPLEMENTARY MATERIAL**

Supplementary Method

*Pilot Study by NanoString nCounter Technology*

Twelve serum from 3 patients with central vertigo due to posterior circulation stroke and 3 patients with peripheral vertigo were collected in the acute phase within 72 hours after onset of vertigo, and at follow-up period on day 90. Potential miRNA candidates were identified from the serum samples by NanoString nCounter System (NanoString Technologies Inc., Seattle, WA, USA). In brief, 100 nanogram of extracted serum miRNA was added into an annealing mastermix which consisted of annealing buffer, the nCounter^®^ miRNA Tag Reagent, and 1:500 dilution of miRNA Assay Controls. Annealing and ligation protocols were conducted according to the manufacturer’s instruction. The purification protocol was initiated after completion of the ligation protocol. A hybridization master mix consisting of the Reporter CodeSet, the Capture ProbeSet, and hybridization buffer was added to a 5 µL of miRNA sample. The hybridization assay was incubated at 65 ºC overnight (12 hours). A Prep Station was set to the high sensitivity protocol. Data was obtained by the nCounter Digital Analyzer. The Charge-Coupled Device (CCD) camera took images of immobilized fluorescent reporters in the sample cartridge with the standard data resolution of 280 fields of view (FOV) per cartridge lane. Normalized counts were obtained from raw counts normalized to an exogenous synthetic spike-in miRNA, cel-miR-254.

Supplementary Result

*miRNA expression profiling in pilot study from NanoString Analysis*

MiRNAs with counts above the average counts of the exogenous spike-in, cel-miR-254, were selected for subsequent analysis. Differential expression of miRNA was assessed by a Venn diagram (1). From the NanoString analysis, the serum miRNAs expressed only in the acute phase in samples of more than one patient with posterior circulation stroke were selected as potential miRNA candidates **(Supplementary Figure I)**.

There were 22 miRNAs expressed only during acute phase in the patients with acute vertigo due to posterior circulation stroke. In order to identify the possible brain specific miRNA candidates, these miRNAs were carefully reviewed in the human miRNA tissue atlas (2). Among these 22, three miRNAs with high level of expression in the brain including miR-342-3p, miR-376-3p, and miR-433-5p were selected for the subsequent validation phase by RT-qPCR.

Supplementary Tables

Table I: Spearman’s correlation coefficients for each miRNA combination

| r_s_ (*P* value) | miR-125a-5p | miR-125b-5p | miR-143-3p | miR-342-3p | miR-376a-3p | miR-433-5p |
| --- | --- | --- | --- | --- | --- | --- |
| miR-125a-5p | 1 |  |  |  |  |  |
| miR-125b-5p | 0.847 (<0.001) | 1 |  |  |  |  |
| miR-143-3p | 0.706 (<0.001) | 0.550 (<0.001) | 1 |  |  |  |
| miR-342-3p | 0.234 (0.077) | 0.167 (0.211) | 0.184 (0.167) | 1 |  |  |
| miR-376a-3p | 0.626 (<0.001) | 0.457 (<0.001) | 0.793 (<0.001) | 0.440 (<0.001) | 1 |  |
| miR-433-5p | 0.686 (<0.001) | 0.536 (<0.001) | 0.736 (<0.001) | 0.138 (0.303) | 0.678 (<0.001) | 1 |

Table II: Univariate and multivariate logistic regression for diagnosis of stroke

|  | Univariate analysis | | | Multivariate analysis✝ | | | | | |  |
| --- | --- | --- | --- | --- | --- | --- | --- | --- | --- | --- |
|  | **Unadjusted OR**  **(95% CI)** | | ***P* Value** | | **Adjusted OR**  **(95% CI)** | | ***P* Value** | |  |  |
| miR-125a-5p | | 1.002 (1.000-1.004) | 0.016* | | | 1.004 (1.001-1.007) | | 0.013* | | |
| miR-125b-5p | | 1.009 (1.002-1.016) | 0.016* | | | 1.021 (1.007-1.035) | | 0.004* | | |
| miR-143-3p | | 1.003 (1.000-1.007) | 0.046* | | | 1.009 (1.002-1.015) | | 0.013* | | |
| miR-342-3p | | 0.999 (0.998-1.001) | 0.323 | | | 1.000 (0.998-1.001) | | 0.81 | | |
| miR-376a-3p | | 1.002 (0.998-1.006) | 0.31 | | | 1.003 (0.998-1.008) | | 0.252 | | |
| miR-433-5p | | 1.008 (1.000-1.016) | 0.044* | | | 1.017 (1.003-1.032) | | 0.016* | | |

**P*<0.05

✝Adjusted for sex, onset to blood collection time, diabetes mellitus, history of myocardial infarction, smoking

Table III: Spearman’s correlation coefficients for the association between miRNA levels and NIHSS

|  | r_s_ *P* value | |
| --- | --- | --- |
| miR-125a-5p | 0.239 | 0.272 |
| miR-125b-5p | -0.006 | 0.977 |
| miR-143-3p | 0.086 | 0.696 |
| miR-342-3p | 0.275 | 0.203 |
| miR-376a-3p | 0.200 | 0.360 |
| miR-433-5p | 0.085 | 0.698 |

**P*<0.05

Table IV: Spearman’s correlation coefficients for the association between miRNA levels and infarction volume

|  | r_s_ *P* value | |
| --- | --- | --- |
| miR-125a-5p | 0.530 | 0.009* |
| miR-125b-5p | 0.546 | 0.007 |
| miR-143-3p | 0.189 | 0.387 |
| miR-342-3p | 0.305 | 0.157 |
| miR-376a-3p | 0.423 | 0.044* |
| miR-433-5p | 0.239 | 0.273 |

**P*<0.05

Table V: Spearman’s correlation coefficients for the association between miRNA level and onset to blood collection time

|  | r_s_ *P* value | |
| --- | --- | --- |
| miR-125a-5p | 0.027 | 0.902 |
| miR-125b-5p | -0.138 | 0.530 |
| miR-143-3p | 0.159 | 0.470 |
| miR-342-3p | 0.030 | 0.891 |
| miR-376a-3p | -0.138 | 0.529 |
| miR-433-5p | 0.318 | 0.136 |

**P*<0.05

Table VI: *P* values of the AUROC comparison between individual miRNA levels and a combination of miRNAs

| *P* value | miR-125a-5p | miR-125b-5p | miR-433-5p |
| --- | --- | --- | --- |
| miR-125a-5p+miR-125b-5p | 0.4682 | 0.9278 |  |
| miR-125a-5p+miR-433-5p | 0.9538 | - | 0.4219 |
| miR-125b-5p+miR-433-5p | - | 0.7402 | 0.3703 |
| miR-125a-5p+miR-125b-5p+miR-433-5p | 0.6443 | 0.8529 | 0.3205 |

Supplement Figures

Figure I: Venn diagram of potential miRNA candidates
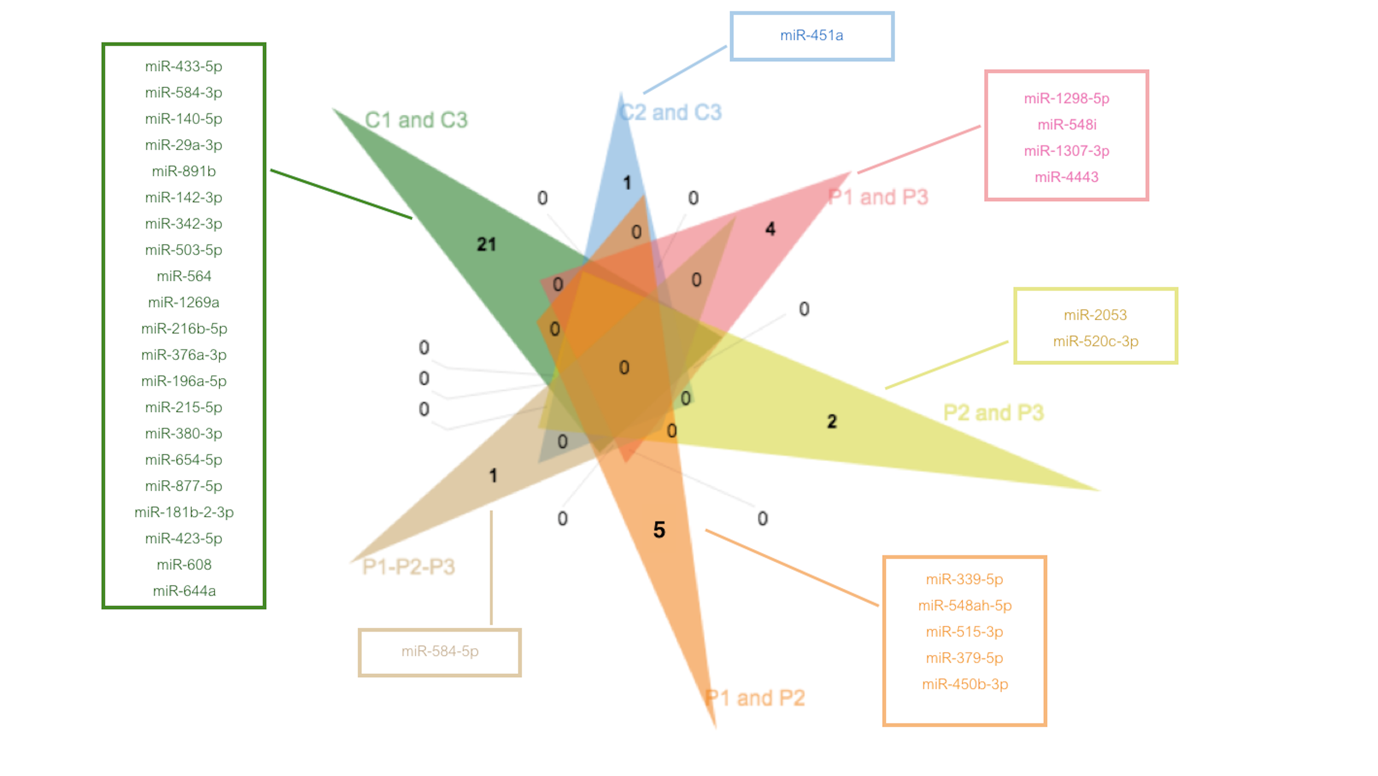


Figure II: AUROC of potential miRNA candidates


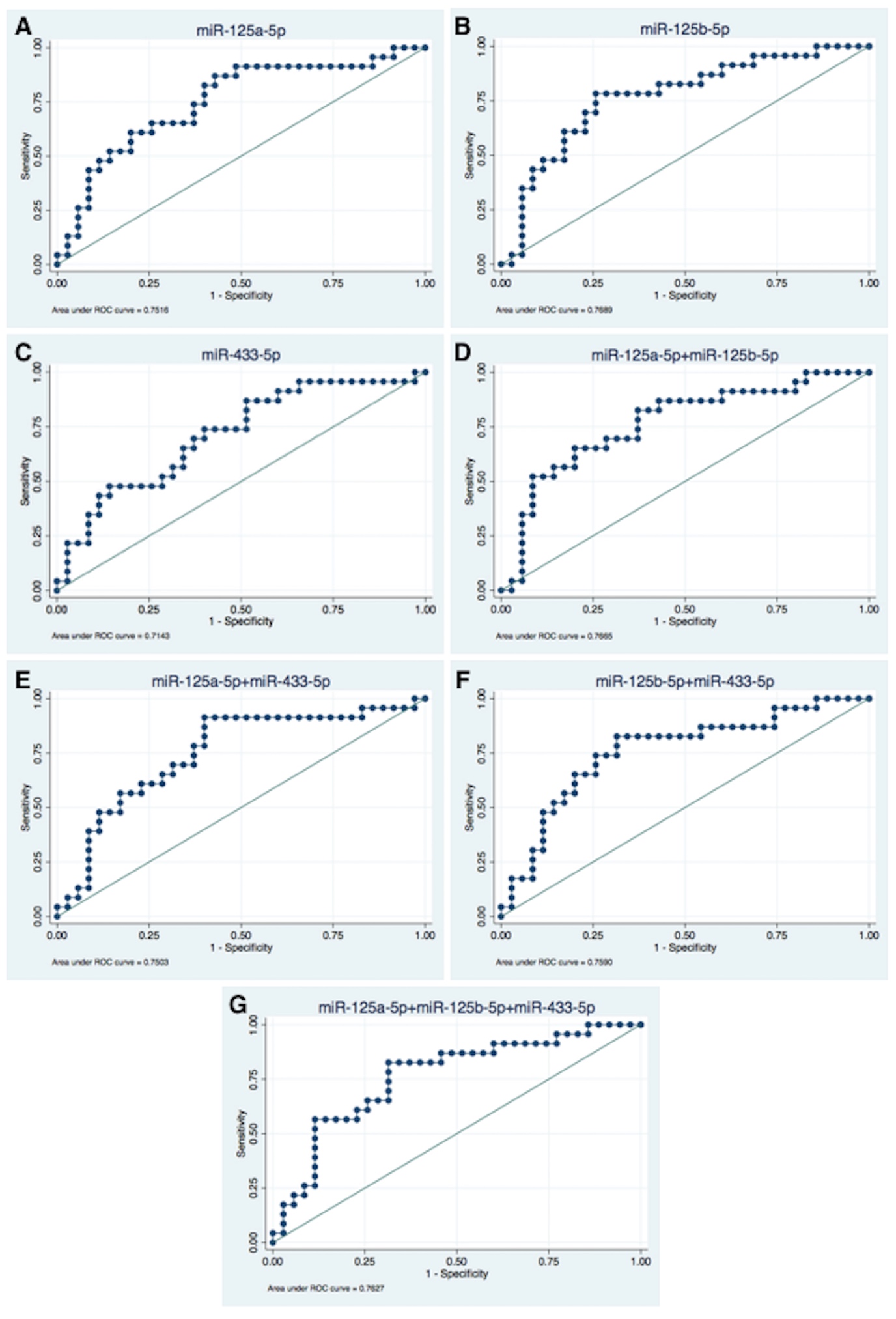


References

1. Bardou P, Mariette J, Escudié F, Djemiel C, Klopp C. jvenn: an interactive Venn diagram viewer. *BMC Bioinformatics* (2014) **15**:293.

2. Ludwig N, Leidinger P, Becker K, Backes C, Fehlmann T, Pallasch C, et al. Distribution of miRNA expression across human tissues. *Nucleic Acids Res* (2016) **44**:3865-77.
